# Fulfilling the taxonomic consequence after DNA Barcoding: *Carychiumpanamaense* sp. n. (Eupulmonata, Ellobioidea, Carychiidae) from Panama is described using computed tomographic (CT) imaging

**DOI:** 10.3897/zookeys.795.29339

**Published:** 2018-11-05

**Authors:** Adrienne Jochum, Bernhard Ruthensteiner, Marian Kampschulte, Gunhild Martels, Jeannette Kneubühler, Adrien Favre

**Affiliations:** 1 Naturhistorisches Museum der Burgergemeinde Bern, 3005 Bern, Switzerland and Institute of Ecology and Evolution, University of Bern, 3012 Bern, Switzerland University of Bern Bern Switzerland; 2 Zoologische Staatssammlung München, 81247 München, Germany Zoologische Staatssammlung München München Germany; 3 Department of Radiology, Universitätsklinikum Giessen und Marburg GmbH-Standort Giessen, Center for Radiology, 35385 Giessen, Germany Universitätsklinikum Giessen und Marburg GmbH-Standort Giessen Giessen Germany; 4 Department of Experimental Radiology, Justus-Liebig University Giessen, Biomedical Research Center Seltersberg (BFS), 35392 Giessen, Germany Justus-Liebig University Giessen Giessen Germany; 5 Department of Entomology III, Senckenberg Research Institute and Natural History Museum, 60438 Frankfurt/M., Germany Senckenberg Research Institute and Natural History Museum Frankfurt/M. Germany

**Keywords:** microgastropoda, museum voucher, tropical ecology, conservation, Panamanian snails, Central America

## Abstract

Five years ago, the Panamanian evolutionary lineage (EL) C12 was uncovered along with four other ELs in an integrative phylogenetic investigation of worldwide Carychiidae. Since EL C12 lacked shell material post-molecular analysis to serve as a museum voucher, it remained undescribed. Now, after recent collection efforts of C12 and the congener, *Carychiumzarzaae* Jochum & Weigand, 2017 at their original Panamanian sites, C12 is morphologically described and formally assigned the name, *Carychiumpanamaense* Jochum, **sp. n.** In sync with recent taxonomic treatment of the genus, computed tomography (CT) is used in this work to differentiate shells of *C.panamaense***sp. n.** from geographically-proximal, Caribbean, North and Central American congeners. Recent material of topotypic *Carychiumjardineanum* (Chitty, 1853) and undamaged *C.zarzaae* were additionally CT-scanned and assessed in the comparative analyses.

## Abstract (es)

Hace cinco años, el linaje evolutivo (LE) panameño C12 fue descubierto junto con otros cuatro LEs en un estudio filogenético integrativo mundial de Carychiidae. El LE C12 permaneció sin ser descrito dado que, después de los análisis moleculares, no había conchas disponibles que sirvieran como material de referencia en museos.

Ahora, después de esfuerzos recientes para colectar C12 y el congénere, *Carychiumzarzaae* Jochum & Weigand, 2017 en los sitios panameños originales, C12 es descrito morfológicamente y se le asigna formalmente un nombre, *Carychiumpanamaense* Jochum, **sp. n.** De acuerdo con el tratamiento taxonómico reciente del género, en este trabajo se emplea tomografía computarizada (TC) para diferenciar conchas de *C.panamaense***sp. n.** de congéneres geográficamente cercanos del Caribe, Norte y Centro América. Además, en el análisis comparativo se escaneó con TC y se examinó material reciente del topotípico *Carychiumjardineanum* (Chitty, 1853) y *C.zarzaae* en buen estado.

## Introduction

**Figure 1.**
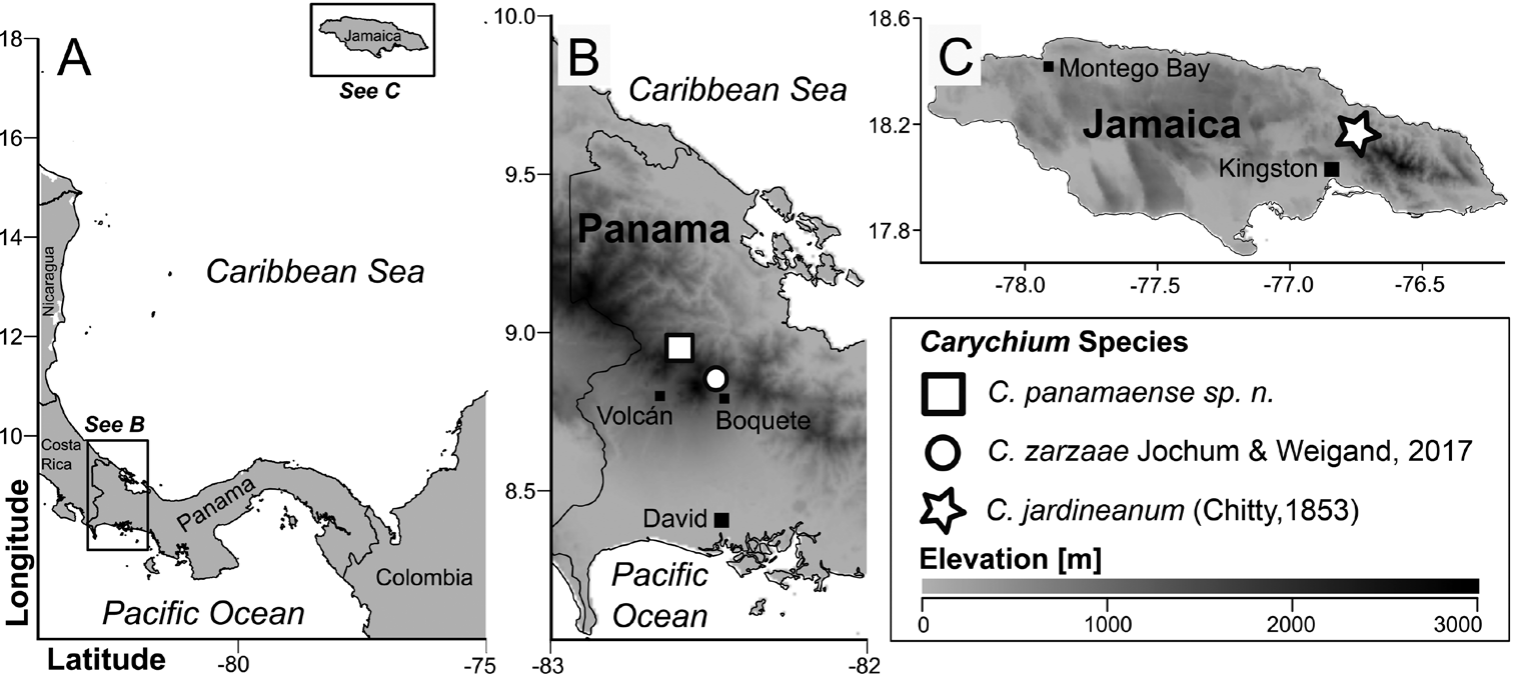
Map indicating type localities of the two Panamanian *Carychium* species, *C.panamaense* sp. n. and *C.zarzaae* Jochum & Weigand, 2017 and of the Jamaican allied species, *C.jardineanum* (Chitty, 1853). The grayscale indicates the local mean elevation. Map downloaded from WORLDCLIM (Hijmans et al. 2005); political borders retrieved from Esri Data and Maps (2002).

In an integrative phylogenetic investigation of worldwide Carychiidae, Weigand et al. (2013) uncovered four evolutionary lineages (ELs) of *Carychium* O.F. Müller, 1773 from North and Central America. These ELs were found to be molecularly distinct from the two known nominal species, *Carychiummexicanum* Pilsbry, 1891 and *C.costaricanum* E. von Martens, 1898. The consequential, morphological and taxonomic assessment of three of these molecularly uncovered lineages resulted in the recent description of three new species of *Carychium* by Jochum et al. (2017): *C.hardiei* Jochum & Weigand, 2017 from Georgia, USA, *C.belizeense* Jochum & Weigand, 2017 from Belize, and *C.zarzaae* Jochum & Weigand, 2017 from Panama. The fourth molecularly flagged EL (C12), also from Panama (Weigand et al. 2013), could not be fully assessed by Jochum et al. (2017, fig. 15) since no shells remained to serve as museum voucher material. Recent collection efforts by one of us (A.F.) at the topotypic locality, Parque International La Amistad in Chiriquí, Panama now enable full taxonomic treatment of lineage C12 (former morphospecies *C.mexicanumcostaricanum* sensu Pilsbry (1948)) in Weigand et al. (2013, fig. 1). In congruence with Jochum et al. (2017), *Carychiumpanamaense* sp. n. is formally described in this work. In addition, since the protoconch and body whorl of the very fragile paratype material of Panama’s recently described congener, *C.zarzaae* (NMBE 549927/1) was damaged in the initial CT-scanning process (Jochum et al. 2017, fig. 14), new images of the fresh topotypic material are presented here in the comparative analysis. Furthermore, although Jochum et al. (2017) presented the distinct molecular aspects of *C.jardineanum* (Chitty, 1853), the only known Caribbean (Jamaica) species of *Carychium*, computer tomographic (CT) images were not available at the time. Their inclusion in the comparative analysis of this work is beneficial for understanding both the spectrum of shell variability and diversity of the tropical American Carychiidae as well as the geographical context of *C.panamaense* sp. n. in particular (Fig. 1).

## Material and methods

*Carychiumpanamaense* sp. n. was collected by A. Favre under the permit Ref. Nr. SE/PH-4-18 issued by the Ministerio de Ambiente, Balboa, Ancón, Panama.

Shell measurements include the shell width (**sw**), shell height (**sh**), aperture width (**aw**) and aperture height (**ah**) expressed in mm (Table 1). Whorl number was counted according to Kerney et al. (1979).

Qualitative aspects of shell morphology include peristome shape; whorl profile (whorl convexity); teleoconch sculpture; development of apertural dentition visible in frontal view; development of the columellar lamella as discernable in the CT images of the ventral, dorsal, side-left and side-right perspectives of the *C.panamaense* sp. n. adult shell.

Material is housed in the following collections:

**AJC** Adrienne Jochum Collection: formerly Institute of Ecology, Evolution & Diversity, Phylogeny & Systematics Collection, Goethe-Universität, Frankfurt am Main, Germany

**ANSP** Academy of Natural Sciences of Drexel University, Philadelphia, PA, USA

**CM** Carnegie Museum of Natural History, Pittsburgh, PA, USA

**MUPADI** Museo de Peces de Agua Dulce e Invertebrados, Universidad Autónoma de Chiriquí, David, Chiriquí, Panama

**NMBE** UNaturhistorisches Museum der Burgergemeinde Bern, Bern, Switzerland

**RBINS** Royal Belgian Institute of Natural Sciences, Brussels, Belgium

**SMF** Forschungsinstitut und Naturmuseum Senckenberg, Frankfurt am Main, Germany

**UF** University of Florida, Florida Museum of Natural History, Gainesville, FL., USA

**Table 1.** Measurement data of *Carychiumpanamaense* sp. n., N=7. Abbreviations: sw – shell width, sh – shell height, aw – aperture width, ah – aperture height. All measurements in millimeters (mm).

***Carychium* specimen**	**Museum No.**	**Sample**	**sw**	**sh**	**aw**	**ah**
*C.panamaense* sp. n. holotype	NMBE 554428	1	0,91	2,12	0,70	0,80
*C.panamaense* sp. n. paratype	NMBE 554429	1	0,84	NA	0,64	0,77
*C.panamaense* sp. n. paratype	NMBE 554429	2	0,76	NA	0,60	0,71
*C.panamaense* sp. n. paratype (damaged)	NMBE 554429	3	NA	NA	NA	NA
*C.panamaense* sp. n. paratype, EtOH	NMBE 554432	1	0,93	2,03	0,68	0,73
*C.panamaense* sp. n. paratype, EtOH	NMBE 554432	2	0,91	1,98	0,69	0,77
*C.panamaense* sp. n. paratype, EtOH	NMBE 554432	3	0,97	2,09	0,74	0,83
**Mean *C.panamaense***			0,89	2,06	0,67	0,77

### Image acquisition

**Figure 2.**
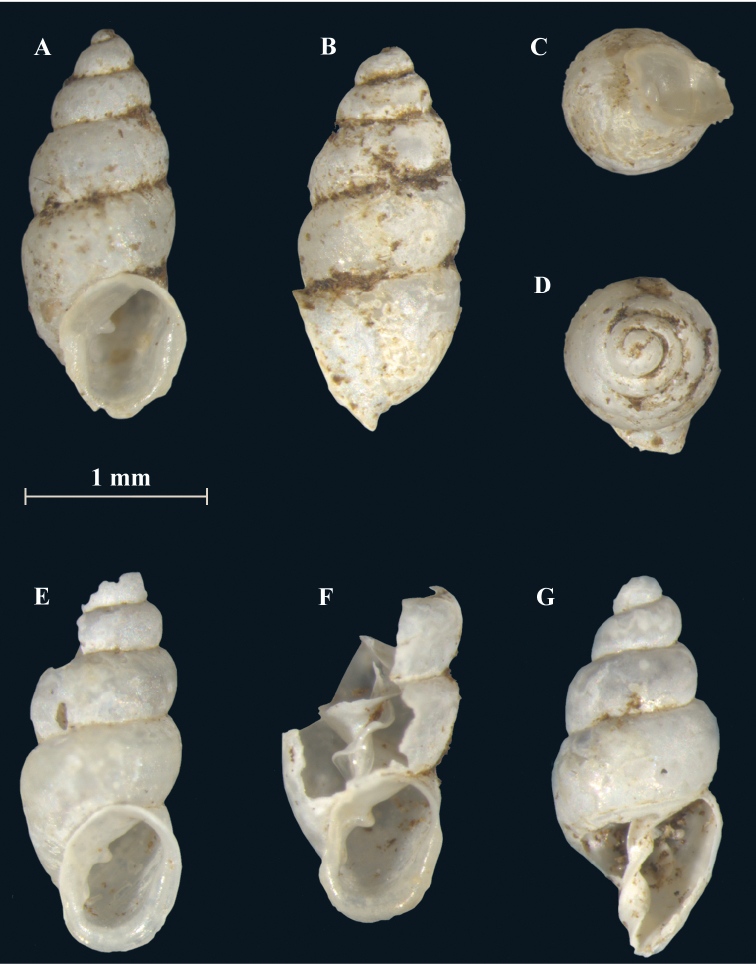
*Carychiumpanamaense* sp. n. **A–D** holotype (NMBE 554428/1) **E–G** paratype shells (NMBE 554429/3). Scale bar: 1 mm.

**Figure 3.**
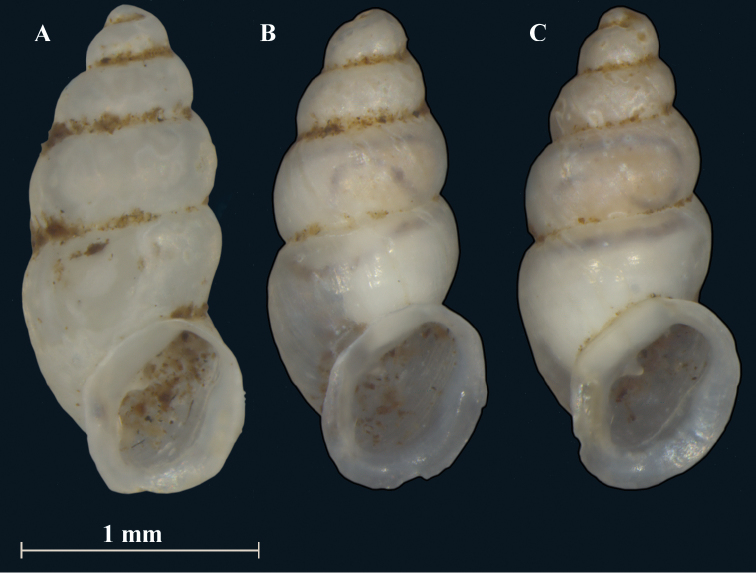
*Carychiumpanamaense* sp. n. **A–C** paratype shells preserved in alcohol (NMBE 554432). Scale bar: 1 mm.

**Digital images**: *Carychiumpanamaense* sp. n. (Figs 2, 3) was imaged using a Leica DFC425 digital camera attached to a Leica M205 C stereo microscope (Wetzlar, Germany), using IMS Client analysis image system software (Imagic Bildverarbeitungs AG, Glattbrugg, Switzerland) for measurements.

**Figure 4.**
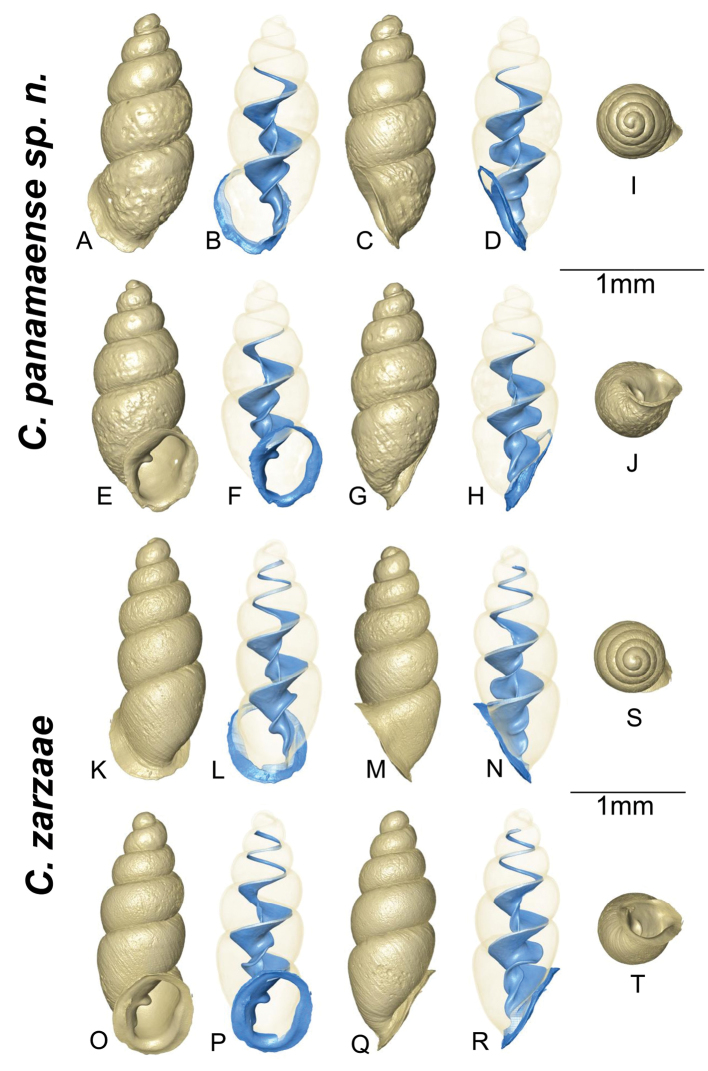
**A–J***Carychiumpanamaense* sp. n. holotype (NMBE 554428/1), CT images, partly with external shell transparent to show columellar apparatus **I** aerial view of protoconch and spire **J** umbilical view **K–T** allied Panamanian species topotype, *Carychiumzarzaae*, Jochum & Weigand, 2017 (AJC 2385). CT images, partly with external shell transparent to show columellar apparatus **S** aerial view of protoconch and spire **T** umbilical view. Scale bar: 1 mm.

**Figure 5.**
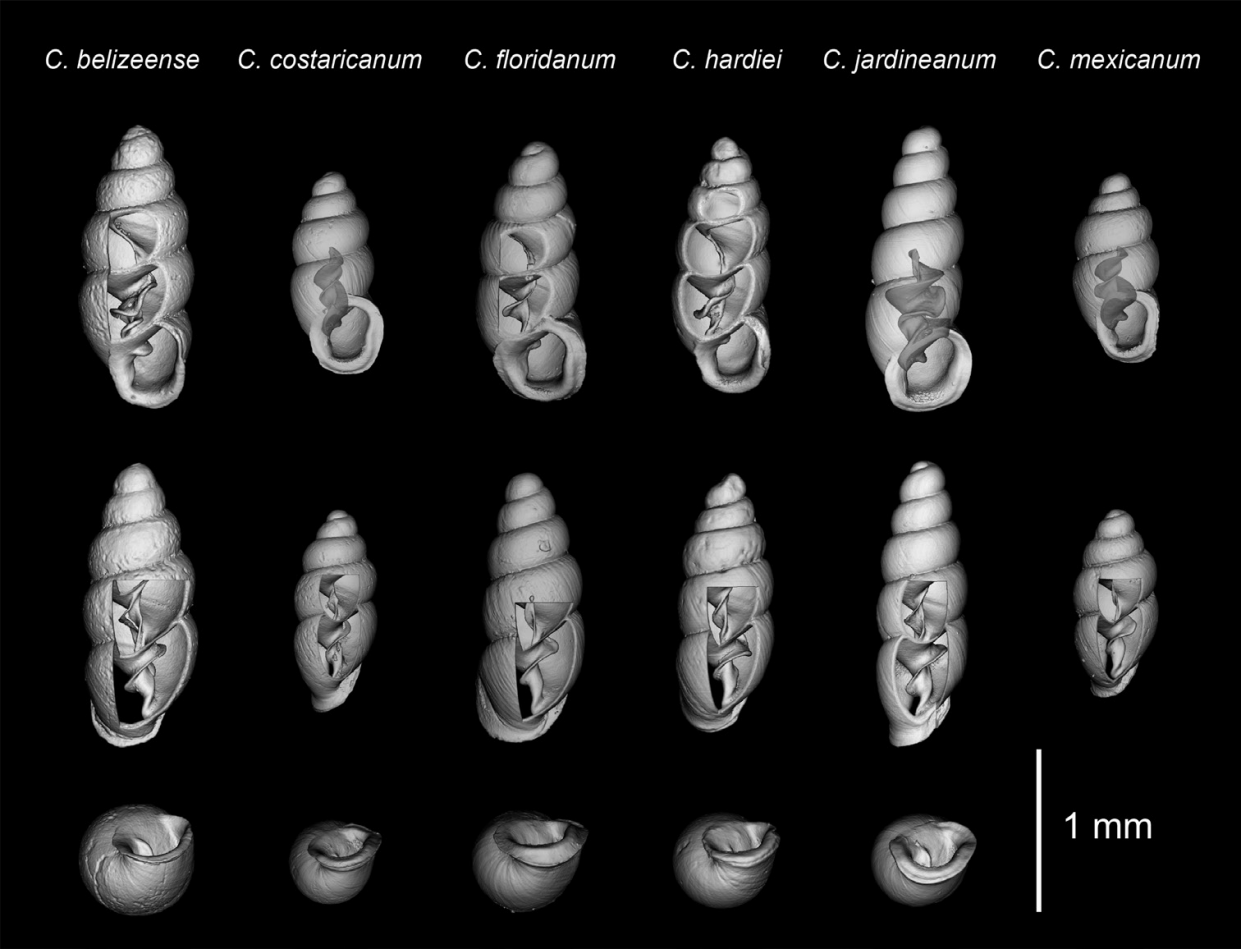
Comparative material (Jochum et al. 2017), *C.belizeense* Jochum & Weigand, 2017 paratype (NMBE 549924/8), *C.costaricanum* E. von Martens, 1898 (RBINS 10591), *C.floridanum* Clapp, 1918 (CM 46540), *C.hardiei* Jochum & Weigand, 2017 paratype (NMBE 549921/8), *C.jardineanum* (Chitty, 1853) (AJC 2321), *C.mexicanum* Pilsbry, 1891 (AJC 2092). CT images showing columellar apparatus, configuration of the columellar lamella and umbilical perspectives showing peristome configuration in allied Central American, Southeast USA and Caribbean species. Scale bar: 1 mm.

**Micro-CT**: The two Panamanian species, *C.panamaense* sp. n. (Fig. 4A–J) and *C.zarzaae* (Fig. 4K–T), were imaged at the Zoologische Staatssammlung München, Munich, Germany. Scanning was performed with a Phoenix Nanotom m (GE Measurement & Control, Wunstorf, Germany) cone beam CT scanner at a voltage of 80 kV and a current of 325 mA using a tungsten (“Standard”) target during a 360° rotation. *Carychiumpanamaense* sp. n. was captured in two longitudinal portions at 1200 projection images each at a total duration of 124 minutes; voxel size was 0.891 μm. *Carychiumzarzaae* was captured at 1600 projections at a total duration of 205 minutes; voxel size was 0.919 μm. The 16-bit data sets, generated by reconstruction, were cropped and converted to 8-bit using VGStudio MAX 2.2 software (Volume Graphics, Heidelberg, Germany). Further visualization procedures were carried out with Amira 6.4 software (FEI Visualization Sciences Group, Burlington MA, USA) applying manual segmentation for discrimination of external and internal shell structures. Final visualization was enabled using the Volume Rendering tool. All grey-colored *Carychium* in the comparative analysis in this work (Fig. 5), except for *C.jardineanum*, were figured in Jochum et al. (2017). In congruence with Jochum et al. (2017), *C.jardineanum* was imaged using a SkyScan 2011 (Bruker MicroCT, Kontich, Belgium) micro-CT system, at the Department of Experimental Radiology, Justus-Liebig University Biomedical Research Center Seltersberg (BFS), Giessen, Germany. The *Carychium* were mounted and scanned 185° around their vertical axis in rotation steps of 0.23° at 80 kV tube voltage and 120 μA tube current. Reconstruction was performed using the Feldkamp cone beam reconstruction algorithm. Image resolution was 1.75 μm isotropic voxel side length with a grey scale resolution of 8 bit. Digital images, post processing and visualization (maximum intensity projection – MIP, volume compositing and summed voxel projection), were displayed using the ANALYZE software package (ANALYZE 11.0, Mayo Clinic, Rochester, MN, USA).

## Taxonomy

### Family Carychiidae Jeffreys, 1830

#### Genus *Carychium* O.F. Müller, 1773

##### Carychium panamaense

Taxon classificationAnimaliaEllobiidaEllobiidae

Jochum sp. n.

http://zoobank.org/C70432C6-2FCD-4F9F-A48D-493E9F9E739D

Figures 2 , 3 , 4

Carychium panamaense : Weigand et al., 2013: 3, fig. 1 48|C12; Seq. ID: BARCA142-12, BARCA143-12, BARCA144-12

###### Material examined.

Holotype (NMBE 554428/1 ex AJC 2383): Panama, Chiriquí Prov., Cerro Punta, La Amistad International Park, El Retoño Trail, near Las Nubes Ranger Station; 8.8934278°N, 82.6190528°W, elev. 2239 m, on moist broadleaf litter and twigs; 27 February 2018; leg. Adrien Favre.

Paratypes: locus typicus 3 damaged shells (NMBE 554429/3 ex AJC 2383); 7 specimens in ethanol (NMBE 554432 ex AJC 2382); 5 specimens in ethanol (SMF 349423 ex AJC 2382); 5 specimens in ethanol (MUPADI-Mol.-01-001 ex AJC 2382); 4 specimens in ethanol (ANSP A476441 ex AJC 2382); 5 specimens in ethanol (CM 159907 ex AJC 2382); 3 specimens in ethanol (UF 511987 ex. AJC 2382); data as for holotype.

###### Diagnosis.

Shell ca. 2 mm in height, transparent, elongate-pupiform with an oblique, ovate-shaped and unequally thickened peristome, with a palatal callus, pronounced parieto-columellar callus and a prominent parietal denticle. Internal coiling of the lamella about the columellar spindle is wide rather than tight.

###### Description.

Measurements are provided in Table 1. Shell minute, elongate pupiform, transparent when fresh, with about 4.1 convex whorls and a deeply incised suture; occasional, irregular striations or growth lines on the body whorl (see also Jochum et al. 2017, fig. 15). The shell is opaque with age and often superficially degraded with pock marks (due to acidity of the leaf litter). The protoconch is more nipple-like than bulbous. The teleoconch is smooth. Peristome is obliquely auriform, longer than wide, tending to be thinnest on the upper right-hand margin, where it slightly reflects from the body whorl and then curves into a relatively broad, shield-like aspect onto the body whorl (Figs 2A, E, 3A). The peristome is otherwise, uniform in thickness (Figs 2, 3) but becomes thinner towards the edges. A medium-sized parietal denticle is present, the base of which is in line horizontally with the widest, shield-like extension of the peristome onto the body whorl (Figs 2A, E, 3, 4F). Directly opposite the parietal denticle is a thickened palatal callus (Figs 2A, 4F). The lower left columellar margin has a prominently-thickened, parietal-columellar callus (Figs 2A, E, F, 3A–C, 4F). In aperture facing-right perspective, the peristome is sheer with the body whorl (Fig. 4G–H). The peristome curves back slightly at the base (Figs 2B, 4C) whereby, the layer of callus on the palatal side forms a small knob on the rim in the aperture facing-left (Fig. 2B) and dorsal (Fig. 4B) perspectives.

**Figure 6.**
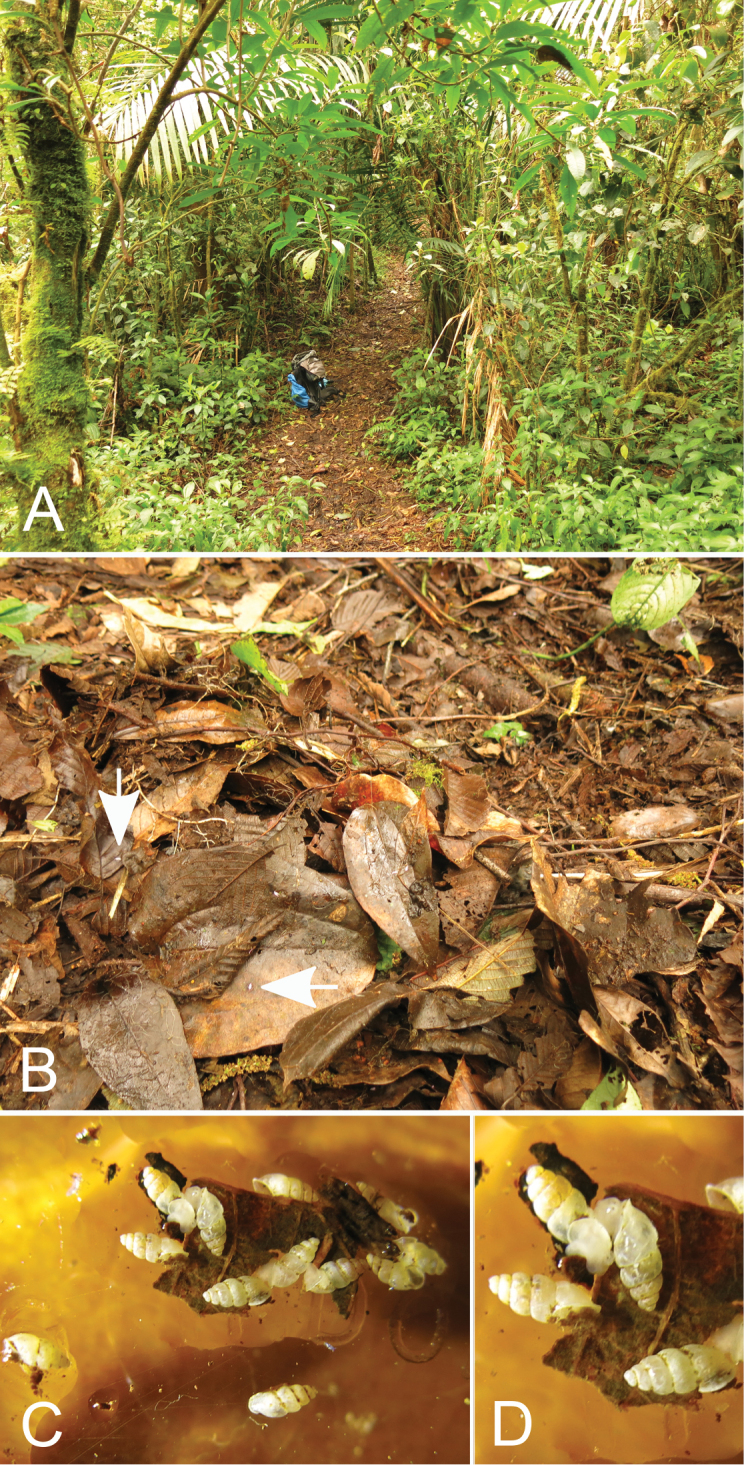
Type locality of *Carychiumpanamaense* sp. n., **A** El Retoño Trail, La Amistad International Park, Cerro Punta, Chiriquí Prov., Panama **B** broadleaf forest litter with white arrows indicating *C.panamaense* sp. n. on leaves **C–D** close-up view of live individuals crawling on leaf.

Internally, a widely spiraling, sinuous lamella starts at the top of the penultimate whorl (dorsal perspective) (Fig. 4B), which extends laterally in aperture facing-left and aperture facing-right perspectives (Fig. 4D, H). The degree of fullest sinuosity varies in the configuration of the lower primary lamella from an accentuated, oblique-elongated S-form (Fig. 2F) to a slightly curved aspect in the ventral perspective (Fig. 4F). The thick, upper curvature of the lamella forms the upper part of the elongated S shape (Fig. 2F). The general curvature of the lamella about the columella is wider than narrow along the entire length of the columellar spindle. Viewed from the umbilical perspective (Fig. 4J), the rim of the peristome is thin and widely flared. In live individuals, the outmost edge of the peristome appears white (Fig. 6D).

###### Differential diagnosis.

Differs from congeners presented in Jochum et al. (2017), imaged here (Figs 4K–T, 5), by its apertural morphology and large apertural size: long, obliquely-auriform, widely-flared aspect of the thinly-rimmed peristome (seen best from umbilical perspective) (Fig. 4) and the wide coiling of the lamella about the columellar spindle. Although the peristome mostly resembles that of *C.belizeense* (Jochum et al. 2017, fig. 11A, I), the generally broad, shield-like extension of the peristome onto the body whorl differentiates this species from *C.belizeense* as well as from its Southeastern USA, Caribbean and Central American congeners. Though the S-shaped configuration of the primary lamella (ventral view) (Fig. 2F) is closest to that of *C.belizeense* (Fig. 5), *C.hardiei* (Fig. 5) and *C.zarzaae* (Fig. 4P), the abapical onset of the lamella in the penultimate whorl and the general extant of sinuosity along the entire length of the columella in relation to the columellar spindle is unique to each species in both the ventral and dorsal perspectives. The tongue-like flexion of the primary lamella is a specific configuration occurring in three different perspectives within the shell of each of these species: *C.hardiei*(dorsal perspective) (Fig. 5), *C.zarzaae* (aperture side-left perspective) (Fig. 4R) and *C.jardineanum* (ventral perspective) (Fig. 5). This down-turned, tongue-like flexion is not at all present in *C.panamaense* sp. n. (Fig. 4). The configuration of the lamella in *C.panamaense* is spatulate (Fig. 4H) rather than tongue-like in form (Fig. 4R).

DNA barcode data can clearly delineate *Carychiumpanamaense* sp. n. from all other North American, Caribbean and Central American taxa (Weigand et al. 2013, Jochum et al. 2017, fig. 3).

###### Etymology.

The new species is named after Panama, the Central American country of origin.

###### Distribution.

Only known from the type locality along the short distance, Retoño trail, ca. 50 m before the first river crossing, Parque International La Amistad, Chiriquí Prov., Panama.

###### Ecology.

In moist broadleaf forest litter and twigs (*Quercus* and Lauraceae) at the base of trees and palm trees in secondary tropical rainforest (Fig. 6A–B).

###### Conservation.

In the flat area of the Retoño trail, where water accumulates under trees during rainfall, live *Carychiumpanamaense* sp. n. was found in relative abundance, suggesting that it has optimum ecological conditions to survive there. *Carychiumpanamaense* sp. n. is only known from Parque International La Amistad, Chiriquí, Panama, a Bi-National Biosphere Reserve (RBA) located between Panama and Costa Rica and designated a UNESCO World Heritage Site. Despite its being found in a Biosphere Reserve, on a global scale, its current distribution may well be limited to the immediate area of Retoño trail. In conjunction with the Guidelines for the IUCN Red List (IUCN Standards and petitions Subcommittee 2014), it is considered a Critically Endangered narrow range endemic (CR B1) and as such, warrants immediate conservation priority.

###### Remarks.

The type locality of the first recorded species of *Carychium* in Panama, *C.zarzaae* (Boquete), is approximately 97 km southeast of the type locality of *C.panamaense* sp. n. near the Las Nubes Ranger Station (Chiriquí). From the site of its closest known Central American relative, *C.costaricanum* (San Gerardo de Dota, San José, Costa Rica) (Weigand et al. 2013), the distance is 263 km.

## Supplementary Material

XML Treatment for Carychium panamaense
